# Comparison of competitive exclusion with classical cleaning and disinfection on bacterial load in pig nursery units

**DOI:** 10.1186/s12917-016-0810-9

**Published:** 2016-09-06

**Authors:** K. Luyckx, S. Millet, S. Van Weyenberg, L. Herman, M. Heyndrickx, J. Dewulf, K. De Reu

**Affiliations:** 1 Institute for Agricultural and Fisheries Research (ILVO), Technology and Food Science Unit, Melle, Belgium; 2 Institute for Agricultural and Fisheries Research (ILVO), Animal Sciences Unit, Melle, Belgium; 3 Department of Pathology, Bacteriology and Poultry Diseases, Ghent University, Faculty of Veterinary Medicine, Merelbeke, Belgium; 4 Veterinary Epidemiology Unit, Department of Reproduction, Obstetrics and Herd Health, Faculty of Veterinary Medicine, Ghent University, Merelbeke, Belgium

**Keywords:** Competitive exclusion, Cleaning and disinfection, Bacterial load, Pig nursery units

## Abstract

**Background:**

Colonisation of the environment of nursery units by pathogenic micro-organisms is an important factor in the persistence and spread of endemic diseases in pigs and zoonotic pathogens. These pathogens are generally controlled by the use of antibiotics and disinfectants. Since an increasing resistance against these measures has been reported in recent years, methods such as competitive exclusion (CE) are promoted as promising alternatives.

**Results:**

This study showed that the infection pressure in CE units after microbial cleaning was not reduced to the same degree as in control units. Despite sufficient administration of probiotic-type spores, the analysed bacteria did not decrease in number after 3 production rounds in CE units, indicating no competitive exclusion. In addition, no differences in feed conversion were found between piglets raised in CE and control units in our study. Also, no differences in faecal consistency (indicator for enteric diseases) was noticed.

**Conclusion:**

These results indicate that the CE protocol is not a valuable alternative for classical C&D.

## Background

Colonisation of the environment in nursery units by pathogenic micro-organisms is an important factor in the persistence and spread of endemic diseases in pigs and of zoonotic pathogens. These infections are often controlled by the use of antibiotics and disinfectants [1]. However, an increasing level of resistance against these substances has been observed in recent years [2–5]. Since 2005, methicillin resistant *Staphylococcus aureus* sequence type 398 (MRSA ST398) has been found on farms and farm animals, especially pigs [6–8]. MRSA ST398 has a multiresistant phenotype [9], a zoonotic character [10] and can also pick up new resistance genes [11]. Wong et al. [12] described the presence of disinfectant resistance genes in porcine MRSA. The minimum inhibitory and bactericidal concentrations (MIC and MBC) of these MRSA strains were lower than the recommended concentrations of disinfectants. However, there is concern that an impairment of the used disinfectant, resulting in exposure to lower active levels of these agents (e.g., due to presence of organic material), resistant MRSA strains harbouring these disinfectant resistance genes may be selected [12]. Slifierz et al. [13] showed that the use of quaternary ammonium compound-based (QAC) disinfectants is a risk for selecting (antibiotic resistant) MRSA in commercial swine herds. Antibiotic multiresistant *Salmonella* strains on pig farms have been described in several countries [14–16]. Randall et al. [17] suggested that the use of biocides alone or combined with antibiotic treatment may also increase selective pressure towards antibiotic resistance of *Salmonella enterica*. Beier et al. [18] showed that β-haemolytic enterotoxigenic *Escherichia coli* (*E. coli*) strains isolated from neonatal pigs, were resistant to chlorhexidine and QAC. Some of these resistant strains had also multiple antibiotic resistance.

Because of the ongoing concern about excessive use of biocides and potential resistance development and cross-resistance to clinically important antibiotics, the use of bacterial biocontrol agents has often been suggested as an alternative method to antagonise the growth of these pathogens. The working mechanism of these biocontrol agents is based on the concept of micro-organisms that should compete with pathogens in the environment by competitive exclusion, influencing quorum sensing, producing antimicrobial compounds (e.g., bacteriocins) and/or competition for attachment sites [19]. However, only very few reports describing the use and the effectiveness of microbial biocontrol agents on farms are available in literature. The aim of this study was to compare the effectiveness of a commercial competitive exclusion (CE) protocol with a classical cleaning and disinfection (C&D) protocol in decreasing *Salmonella*; (haemolytic) *E. coli*, faecal coliforms, *Enterococcus* spp. and MRSA contamination of nursery units during 3 successive rounds.

## Methods

### Management in control and CE units

This study was carried out in 6 identical nursery units at the experimental pig farm of the Institute for Agricultural and Fisheries Research (ILVO) during 3 successive production rounds. Piglets were moved to these units immediately after weaning (4 weeks of age) and stayed there for 6 weeks. Three units were assigned to the control group (classical C&D protocol) and 3 to the treatment group (CE protocol). Each compartment consists of eight identical pens of 1.8 m^2^. Piglets were raised per six in one pen. After 6 weeks, piglets were transported to fattening units and pens were cleaned (and disinfected) according to the tested protocols.

Classical C&D protocol was carried out after pigs were removed. Manure was removed by cleaning with cold water. Twenty-four hours later, pens were soaked with 2 % MS Topfoam (sodium hydroxide) (Schippers, Bladel, The Netherlands) for 30 min. The cleaning product and any remaining dirt was removed under high pressure with cold water (150 bar) and pens were disinfected with 1 % (v/v) MS Megades (glutaraldehyde and quaternary ammonium compounds) (Schippers). Finally, the pens were kept empty during two weeks of down-time.

The CE units pens were first hosed down with cold water to remove manure; 24 h later they were soaked with 1.5 % (v/v) PIP AHC (Probiotics In Process Animal House Cleaner, Chrisal, Lommel, Belgium) at 40 °C for 10 min and rinsed with warm water (40 °C). PIP AHC consists of cleaning compounds, *Bacillus* spp. spores and enzymes. In CE units, no disinfection was carried out. In addition, during the 2-week down-time period as well as during production, CE units were sprayed 2–3 times per week with pure PIP AHS (Animal Housing Stabilizer, Chrisal) to bring and retain biocontrol agents into the stall environment. In the first week of production during the third round, CE units were sprayed every day of the week with PIP AHS. The AHC and AHS PIP products contained *Bacillus* spp. spores of five different species in a concentration of 8.5 and 7.5 log colony forming units (CFU)/mL, respectively.

Both protocols were carried out according to the manufacturers guidelines. For each protocol an individual and identical high pressure jet (Kärcher, HDS 6/14-4CX, Temse, Belgium) was used.

### Sampling scheme

Sampling was performed at different time points (“sampling moments”): (1) immediately after pig loading (before cleaning, BC); (2) 24 h after cleaning (CE units) (AC) or 24 h after disinfection (control units) (AD); (3) after 1 week (W1) and (4) after 5 weeks of production (W5) (piglets present). Three pens per compartment were sampled at each sampling moment. Premoistened sponge swab samples with 10 mL Buffered Peptone Water (BPW) (3 M, SSL10BPW, St-Paul, USA) were taken at five locations per pen: synthetic grid floor, concrete wall, synthetic wall, drinking nipples and feeding trough. Samples were taken in triplicate per type of location resulting in 15 swab samples per nursery unit at each sampling moment. After disinfection, 10 mL Dey Engley neutralising broth (Sigma Aldrich, Fluka, D3435, St-Louis, USA) was used to premoisten the sponge swab samples (SSL100, 3 M) used. A surface of 625 cm^2^ (A4 paper format) was sampled. Because the surface of the drinking nipples was smaller than 625 cm^2^, 2 drinking nipples per pen were sampled and pooled as one sample.

### Sample processing

Samples were transported to the lab under refrigeration and stored at 3 ± 2 °C for 18 h before further processing. Samples were first diluted with 30 ml of BPW (Oxoid, CM0509, Basingstroke, Hampshire, England) and then homogenized by placing them in a Masticator (IUL instruments, S.A., Barcelona, Spain). Prior to plating, swab samples were further serial diluted (1:10) in peptone physiological salt water (Bio Trading, K110B009AA, Mijdrecht, The Netherlands) to produce countable results on the selected agar media: Slanetz-and-Bartley (Oxoid, CM0377) for *Enterococcus* spp., Rapid *E. coli* (Biorad, 356–4024, Marnes-la-Coquettes, France) for *E. coli* and faecal coliforms and chromID® MRSA-SMART (MRSM, bioMérieux, 413050,Marcy l’Etoile, France) for MRSA enumerations. Slanetz and Bartley, Rapid *E. coli* and MRSA-SMART agar plates were incubated at 37 °C during 48 h, 44 °C during 24 h and at 37 °C during 24–48 h, respectively. A 3 ml BPW-fraction of the sample was heated for 10 min at 80 °C, diluted in peptone water and plated on Plate Count Agar (Oxoid, CM0325) for spore enumerations in order to determine the CFU count in both PIP products and to test if *Bacillus* spp. spores were well distributed and sufficiently present in pens. Plate Count Agar plates were incubated for 72 h at 30 °C. Also, a 10 ml BPW-fraction of the sample was mixed with 10 ml double concentrated Mueller Hinton Broth (Oxoid, CM0405) and 13 % (w/v) sodium chloride (Merck, 1.06404.500, Darmstadt, Germany). After overnight incubation at 37 °C, 100 μl was plated on MRSM for detection of MRSA. In addition, the original sample diluted in BPW (i.e., the remaining BPW fraction) was overnight incubated at 37 °C for detection methods. Detection of *E. coli* and faecal coliforms was carried out by plating 10 μl of the enriched BPW fraction on Rapid *E. coli* medium. *Salmonella* detection on the broth was carried out according to ISO 6579:2002 Annex D protocol [20].

### Confirmation of, MRSA, *Salmonella* and haemolytic *E. coli*

Five positive MRSA colonies (if present) were subcultured on Tryptone Soy Agar (Oxoid, CM0131) and DNA was extracted according to the method of Stranden et al. [21]. A multiplex PCR, as described by Maes et al. [22], was performed for MRSA and a CC398 specific PCR, as described by Stegger et al. [23], for MRSA ST398 confirmation.

Positive *Salmonella* colonies on Xylose Lysine Deoxycholate agar medium (Oxoid, CM0469) were subcultured on Nutrient Agar (Oxoid, CM0003). After incubation, PCR confirmation on cel lysates was performed as described by Aabo et al. [24].

From the third down-time and production round, five positive *E. coli* colonies (when possible) were subcultured on Columbia base Blood Agar (Oxoid, CM0331) with 5 % sheep blood and incubated for 24 h at 37 °C for analysis of haemolytic *E. coli*. If a plate was negative after 24 h, it was incubated for a further 24 h. To calculate the enumerations of haemolytic *E. coli*, the ratio of the number of positive haemolytic *E. coli* colonies on the 5 selected colonies was multiplied by the mean *E. coli* enumeration of that sample.

### Other analyses

Piglets were weighed individually at the age of 4, 6 and 9 weeks. Also feed intake was monitored per pen on the same moments allowing to calculate feed conversion ratio of every pen.

In addition, faecal consistency was evaluated according to [25]: a score from 1 (no diarrhea) to 4 (serious diarrhea) was assigned per pen.

Finally, clinical manifestations and treatment with antibiotics were registered. Treatments days per 100 days at risk (TD100) was calculated per pen for each protocol. This was done by calculating the ratio of treatments days (number of days that piglets received antibiotics) and the number of days at risk (time that pigs could be exposed to antibiotics), taking the number of dead piglets into account. This ratio was then multiplied by 100.

### Statistical analysis

The distribution of the dependent variables was characterised with a histogram and Q-Q plot. Log transformed enumerations of spores and *Enterococcus* spp. and results of average daily gain, daily feed intake, feed conversion ratio and TD100 ratio followed a normal distribution. Log transformed enumerations of *E. coli*, haemolytic *E. coli*, faecal coliforms and MRSA did not follow this distribution.

The 4 point scale faecal consistency score was reduced to a binary scale: 0 = pens with score 1 and 1 = pens with score > 1.

The effect of the predictor variables on the normal distributed data (dependent variables) was assessed using multivariate linear regression. The effect of predictor variables on the non normally distributed outcome variables describing the enumeration and detection of the different bacteria (absence or below the detection limit =0, presence =1) was tested by means of multivariate logistic regression analysis.

A backward stepwise elimination was performed to determine the final statistical model for each bacteriological parameter, starting with the global model (predictor variables: protocol used, sampling moment, production round and location) and subsequently removing all non-significant terms. Only biologically relevant interaction effects were considered. In each model, the variables compartment and pen were included as a random effect to correct for measurements within one pen and compartment. The predictor variable sampling moment was included as a repeated measure. Post-hoc comparison was performed with a Tukey-Kramer test. Throughout the analyses, *P*-values ≤ 0.05 were considered as significant.

All statistical analyses were carried out with Statistical Analysis System software (SAS®, version 9.4, SAS Institute Inc.).

## Results

In total 1074 swab samples were taken during 3 successive rounds. At each sampling moment approximately 90 samples were taken: i.e., 45 in CE units (*n* = 3) and 45 in control units (*n* = 3).

### Spore enumerations

**Fig. 1:**
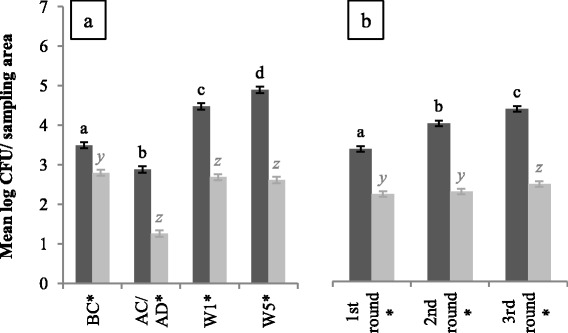
Mean spore enumerations in log colony forming units/sampling area for CE (*dark grey bars*) and control units (*light grey bars*). At each sampling moment (**a**) and per round (**b**), 135 and 180 samples were taken per unit type, respectively. Significant differences between sampling moments or rounds within one type of unit are indicated by different letters above bars. Significant differences between protocols within one sampling moment or round are indicated by a star (*) on the horizontal axis. Vertical bars denote standard errors. BC, before cleaning; AC/AD, after cleaning (CE unit) or after disinfection (control unit); W1, after 1 week of production; W5: after 5 weeks of production

At every sampling moment and in each production round, higher spore enumerations were found for CE units compared to control units (*P* < 0.01) (Fig. 1a and b), with a minimal difference of 0.70 log (BC) and 1.15 log (first round) CFU (colony forming units)/sampling area. Further, spore enumerations increased after every round in CE units (*P* < 0.01) (Fig. 1b). Mean spore enumerations ranged from 2.88 log CFU/sampling area AC to 4.89 log CFU/sampling area at W5 during production piglets present and from 1.25 log CFU/sampling area AD to 2.61 log CFU/sampling area at W5 for CE and control units, respectively.

### *Enterococcus spp*. enumerations

**Fig. 2:**
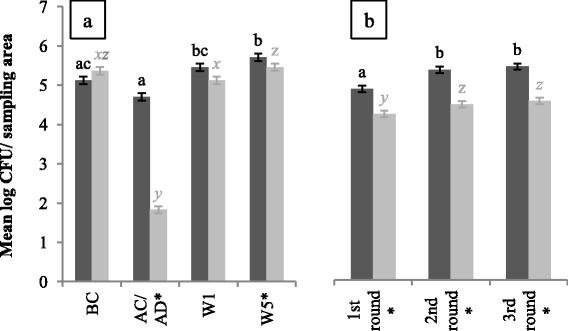
Mean *Enterococcus* spp. enumerations in log colony forming units/sampling area for CE (*dark grey bars*) and control units (*light grey bars*). At each sampling moment (**a**) and per round (**b**), 135 and 180 samples were taken per unit type, respectively. Significant differences between sampling moments or rounds within one type of unit are indicated by different letters above bars. Significant differences between protocols within one sampling moment or round are indicated by a star (*) on the horizontal axis. Vertical bars denote standard errors. BC; before cleaning, AC/AD, after cleaning (CE unit) or after disinfection (control unit); W1, after 1 week of production and W5: after 5 weeks of production

When considering the overall contamination level in both units, higher *Enterococcus* spp. enumerations, with a mean difference of 0.80 log CFU/sampling area, were found in CE units (*P* < 0.01). After disinfection of control units, lower *Enterococcus* spp. enumerations were observed compared to cleaned CE units (*P* < 0.01) (Fig. 2a). The mean difference was 2.88 log CFU/sampling area. Cleaning of CE units caused a reduction of 0.42 log CFU/sampling area, while in disinfected control units a reduction of 3.54 log CFU/sampling area was noticed. Before cleaning and after 1 week of production, no differences in *Enterococcus* spp. enumerations were found between units. However, at W5, higher *Enterococcus* spp. enumerations were found in CE units (*P* = 0.05). In addition, *Enterococcus* spp. enumerations were higher in every production round for CE units (*P* < 0.01) (Fig. 2b).

### *E. coli* enumerations

**Fig. 3:**
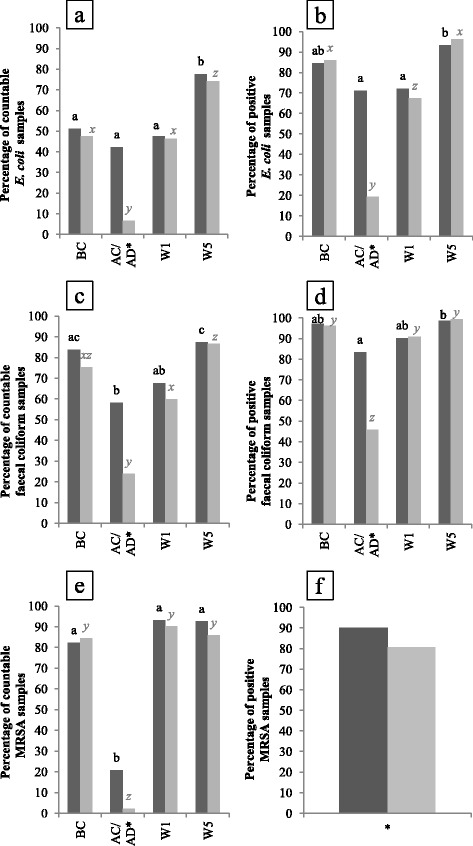
Percentage of positive samples before (enumerations) and after enrichment (detection) for *E. coli * (**a**-**b**), faecal coliforms (**c**-**d**) and MRSA (**e**-**f**) given for CE (*dark grey bars*) and control units (*light grey bars*). At each sampling moment and in total 135 and 540 samples were taken per unit type, respectively. Significant differences between sampling moments within one type of unit are indicated by letters above bars. Significant differences between protocols within one sampling moment are indicated by a star (*) on the horizontal axis. BC, before cleaning; AC/AD, after cleaning or after disinfection; W1, after 1 week of production and W5: after 5 weeks of production

More *E. coli* countable samples were found for CE units after cleaning compared to control units after disinfection (*P* < 0.01) (Fig. 3a). Proportion of countable samples was reduced by 9 % AC of CE units, while a reduction of 41 % was obtained after disinfecting control units. During production and before cleaning, no differences were found in amount of countable *E. coli* samples between both types of units.

In control units, lower amounts of countable samples were found AD compared to amounts found BC and W1 (*P* < 0.01) while this was not seen AC of CE units (Fig. 3a).

Descriptive values of *E. coli* enumeration at each sampling moment are given in Table 1.

**Table 1:** Descriptive values for *Escherichia coli* (*E. coli*), faecal coliforms and methicillin resistant *Staphylococcus aureus* (MRSA) enumerations (log colony forming units/sampling area) given for each sampling moment for CE units and control units

Sampling moment	*E. coli*	Faecal coliforms	MRSA
CE units			
BC^a^	0.0–**1.6**–2.8	2.7 ± 1.5	2.9 ± 1.4
AC/AD^b^	0.0–**0.0**–2.8	0.0–**1.9**–3.8	0.0–**0.0**–0.0
W1^c^	0.0–**0.0**–2.8	0.0–**2.7**–3.8	3.3 ± 1.1
W5^d^	2.5 ± 1.6	3.1 ± 1.5	3.2 ± 1.1
Control units			
BC	0.0–**0.0**–3.0	2.6 ± 1.7	2.9 ± 1.4
AC/AD	0.0–**0.0–**0.0	0.0–**0.0**–0.0	0.0–**0.0**–0.0
W1	0.0–**0.0**–3.0	0.0–**2.0**–3.6	3.2 ± 1.3
W5	2.5 ± 1.8	3.1 ± 1.6	2.9 ± 1.3

Mean and standard deviation are given for enumerations that are normally distributed. First quartile (Q1), median (Q2, bold cha﻿racters) and third quartile (Q3) are given for enumerations that did not follow this distribution

^a^BC, before cleaning

^b^AC/AD, after cleaning/after disinfection

^c^W1, after 1 week of production

^d^W5, after 5 weeks of production

### Haemolytic *E. coli* enumerations

Of all samples taken in CE units (*n* = 180) and control units (*n* = 180) during the 3rd round, 24 % and 23 % were positive for haemolytic *E. coli*, respectively. Of these positive samples, 16 % were obtained AC (CE units) and 0 % were obtained AD (control units), respectively. Mean enumerations were 3.0 log CFU/sampling area for both types of units. No significant differences were noticed between units.

### Faecal coliform enumerations

When comparing CE and control units, results of faecal coliform enumeration confirmed the observations obtained with *E. coli* analyses (Fig. 3c). A reduction of 26 and 51 % of faecal coliform countable samples was obtained AC and AD of CE and control units, respectively.

After cleaning as well as AD, a significant reduction of faecal coliform countable samples was obtained (*P* < 0.01).

Faecal coliform enumerations at each sampling moment for both types of units are given in Table 1.

### *E. coli* and faecal coliform detection

Detection results of *E. coli* (Fig. 3b) and faecal coliforms (Fig. 3d) confirmed the enumeration results of both parameters.

### MRSA enumerations

After cleaning, countable samples were reduced 61 % for CE units, 20 % less than the observed reduction in disinfected control units (*P* < 0.01) (Fig. 3e). When pens were soiled (BC, W1 and W5), no differences in MRSA contamination were found between both types of units.

Mean and median enumerations for MRSA are given for each sampling moment in Table 1.

### MRSA detection

Detection results showed that the number of MRSA positive samples was the highest (90 %) for CE units compared to the control units (81 %) (*P* < 0.01) (Fig. 3f).

### *Salmonella* detection

No *Salmonella* was found in this study.

### Sampling locations

**Fig. 4:**
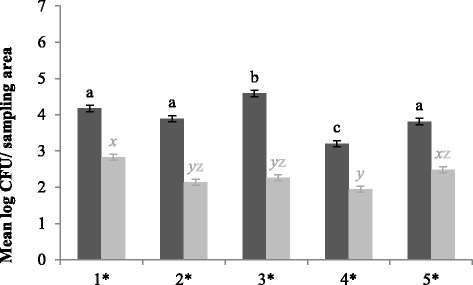
Mean spore enumerations in log colony forming units/sampling area for CE (*dark grey bars*) and control units (*light grey bars*) for each location. At each location, 108 samples were taken per type of unit. Significant differences between sampling moments within one type of unit are indicated by different letters above bars. Significant differences between protocols within one sampling moment are indicated by a star (*) on the horizontal axis. Vertical bars denote standard errors. 1, grid floor; 2, concrete wall; 3, synthetic wall; 4, drinking nipples; 5, feeding trough

**Fig. 5:**
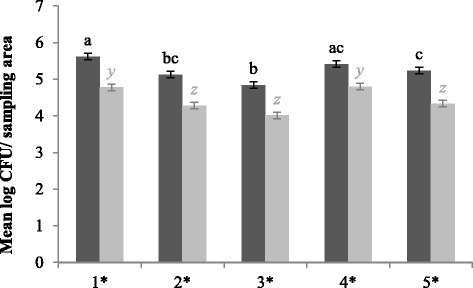
Mean *Enterococcus* spp. enumerations in log colony forming units/sampling area for CE (*dark grey bars*) and control units (*light grey bars*) for each location. At each location, 108 samples were taken per type of unit. Significant differences between sampling moments within one type of unit are indicated by different letters above bars. Significant differences between protocols within one sampling moment are indicated by a star (*) on the horizontal axis. Vertical bars denote standard errors. 1, grid floor; 2, concrete wall; 3, synthetic wall; 4, drinking nipples; 5, feeding trough

Mean enumerations (with standard deviation) and median enumerations (with first and third quartile) of *Enterococcus* spp., *E. coli*, faecal coliforms and MRSA after cleaning (CE units) and disinfection (control units) are given per type of sampling location in Table 2. In addition, the percentage of countable swab samples (enumerations) and positive samples after enrichment (detection) is shown for both types of units. Also, mean spore and *Enterococcus* spp. counts on all samples taken in CE and control units are given for each type of location in Figs. 4 and 5, respectively.

**Table 2:** Descriptive values for *Escherichia coli* (*E. coli*), faecal coliforms and methicillin resistant *Staphylococcus aureus* (MRSA) enumerations (log colony forming units/sampling area) and detection after cleaning (CE units) and disinfection (control units) for each type of sampling location. Detection method was carried out after an overnight enrichment of samples

Location	*Enterococcus* spp.		*E.coli*			Faecal coliforms			MRSA		
	CS (%)^f^	Enumerations	CS (%)	Enumerations	D (%)^g^	CS (%)	Enumerations	D (%)	CS (%)	Enumerations	D (%)
CE units											
1^a^	100	5.0 ± 0.8	59	0.0–**1.6**–3.0	85	67	0.0–**3.2**–3.7	96	44	0.0–**0.0**–1.9	81
2^b^	100	4.8 ± 1.0	67	0.0–**1.6**–4.1	78	90	2.6–**3.9**–4.9	92	22	0.0–**0.0**–0.0	74
3^c^	100	4.4 ± 0.9	4	0.0–**0.0**–0.0	48	19	0.0–**0.0**–0.0	50	11	0.0–**0.0**–0.0	56
4^d^	100	4.9 ± 0.4	41	0.0–**0.0**–3.0	85	52	0.0–**2.5**–3.7	96	19	0.0–**0.0**–0.0	63
5^e^	96	4.4 ± 1.3	41	0.0–**0.0**–2.2	59	62	0.0–**2.5**–3.6	83	7	0.0–**0.0**–0.0	44
Control units											
1	70	2.1 ± 1.6	11	0.0–**0.0**–0.0	26	33	0.0–**0.0**–2.5	58	0	0.0–**0.0**–0.0	26
2	48	0.0–**0.0**–3.0	0	0.0–**0.0**–0.0	4	10	0.0–**0.0**–0.0	46	0	0.0–**0.0**–0.0	19
3	33	0.0–**0.0**–1.7	0	0.0–**0.0**–0.0	7	5	0.0–**0.0**–0.0	17	4	0.0–**0.0**–0.0	19
4	89	3.3 ± 1.5	19	0.0–**0.0**–0.0	30	43	0.0–**0.0**–3.2	67	4	0.0–**0.0**–0.0	37
5	48	0.0–**0.0**–2.9	4	0.0–**0.0**–0.0	30	29	0.0–**0.0**–2.8	42	4	0.0–**0.0**–0.0	15

Mean and standard deviation are given for enumerations that are normally distributed. First quartile (Q1), median (Q2, bold characters) and third quartile (Q3) are given for enumerations that did not follow this distribution

^a^1, floors

^b^2, concrete walls

^c^3, synthetic walls

^d^4, drinking nipples

^e^5, feeding trough

^f^CS (%), proportion of countable samples given in percentage

^g^D (%), proportion of positive samples after detection given in percentage

After cleaning of CE units, enumerations of *Enterococcus* spp. were the highest for floors, concrete walls and drinking nipples. In addition, highest percentage of countable *E. coli* samples and median enumerations were found for floors and concrete walls. Moreover, after enrichment also drinking nipples were still often contaminated with *E. coli*. Results of faecal coliforms and MRSA confirmed these observations.

In control units, high numbers of *Enterococcus* spp. were found on floors and drinking nipples. Most *E. coli* positive samples after enrichment were found for floors, drinking nipples and feeding troughs. In addition, highest enumerations for faecal coliforms were also found at these locations. Finally, for MRSA, drinking nipples were the most contaminated after disinfection.

More spore enumerations were found at every location for CE units (Fig. 4), with a minimal difference of 1.2 log CFU/sampling area.

In addition, when considering the overall *Enterococcus* spp. contamination level, higher levels were found for each location in CE units (Fig. 5).

### Performance results

Mean starting weight of piglets in CE and control pens was 7.4 ± 1.5 and 7.1 ± 1.5 kg, respectively. A mean feed intake of 0.539 ± 0.078 and 0.521 ± 0.065 kg/day was observed for CE and control units, respectively. No significant differences were found between feed intake of piglets raised in CE and control pens. When considering results of daily gain, no significant differences were found. Average daily gain was 0.407 ± 0.056 and 0.395 ± 0.053 kg for piglets in CE and control pens, respectively. In addition, no significant differences in mean feed conversion were found: 1.327 ± 0.072 and 1.324 ± 0.085 for pigs in CE and control units, respectively.

### Faecal consistency

No significant differences in scores of faecal consistency between protocols were noticed (data not shown).

### Antibiotic treatment

The mean TD100 for CE and control units was 27.9 ± 0.9 and 28.3 ± 2.1 %, respectively. No significant differences were found between protocols.

## Discussion

The emergence of multiresistant (pathogenic) bacteria is of great concern for animal and human health. Excessive use of antibiotics [26, 27] and disinfectants [28–30] in for example the animal primary production, could possibly contribute to this phenomenon. Therefore, alternative methods such as competitive exclusion (CE) are promoted as promising. In this study a commercial CE protocol (by probiotic-type bacteria) was compared with a classical C&D protocol in nursery units.

According to the manufacturer of the PIP products, a reduction of pathogenic bacteria and improvement in hygiene after CE during 3 successive production rounds should be obtained. The first statement could not be confirmed by this study: *E. coli* (*Salmonella*-indicator), haemolytic *E. coli* and MRSA analyses showed that the infection pressure after CE cleaning was not reduced to the same extent as implementing a disinfection step. Furthermore, during production no differences were noticed. Also no improvement in hygiene was seen: during the 5th week of production higher *Enterococcus* spp. enumerations (hygiene indicator) and no differences in faecal coliforms (faecal indicator) contamination between the two types of units were found. Because, higher contamination levels of MRSA and pathogen-indicator organisms (*E. coli*) were found in CE units after cleaning, there may be a greater chance of infecting young piglets arriving in those nurseries.

Several hypotheses have been proposed to explain the mechanisms of CE cultures. One is that CE bacteria should compete with other bacteria for adhesion sites, nutrients and energy, which results in preventing growth and proliferation of pathogenic bacteria in the environment (Cummings and Macfarlane, [31]). Another hypothesis is that these bacteria influence the quorum sensing communication and therefore inhibit expression of virulence and colonisation genes of pathogens (Vilà et al. [32]; Deep et al. [33] ). Besides CE bacteria, also enzymes were administered during cleaning, with the aim of helping to eliminate biofilms. In this study, no reduction of the analysed bacteria after 3 production rounds in CE units was seen. Several explanations were found to clarify this observation: (i) adhesion sites are abundantly present in animal houses, hence there is no need for competition; (ii) removal of organic debris is only carried out when piglets are removed from pens, therefore CE-, pathogenic and other bacteria have an abundance of nutrients during production, eliminating the need for competition between bacteria; (iii) however, in order to compete for nutrients, spores need to germinate, which may not be the case for all spores.

Moreover, Luyckx, et al. [34] (i.e., chapter III) showed that a cleaning step in broiler houses caused a reduction of total aerobic bacteria with 2 log CFU/sampling surface and that a disinfection step caused a further reduction of 1.5 log CFU. Although, cleaning caused a greater reduction of total aerobic bacteria, both the above study and this one showed that a disinfection step is still an important step for further reducing the bacterial infection pressure in barns with naturally high levels of environmental bacteria.

Improvement of feed conversion efficiency by probiotic-type bacteria could be obtained by a shift in intestinal flora, stimulating growth of nonpathogenic facultative anaerobic bacteria, inhibiting growth of pathogens, and enhancing digestion and utilisation of nutrients [35]. However, no differences were found between piglets raised in CE and control units in our study. Also, no differences in faecal consistency was noticed. A possible explanation could be that not enough CE bacteria could be administered directly to the animals through the environmental spray application.

Finally, the contamination levels of the different sampling locations were analysed after cleaning of CE units and disinfection of control units. In CE units, grid floors, concrete walls and drinking nipples were still mostly contaminated by *Enterococcus* spp., *E. coli*, faecal coliforms and MRSA after cleaning. Although spore counts showed that high numbers of CE bacteria were present at these locations, the contamination level of different bacteria was still much higher compared to the microbial load after disinfection of control units. In addition, the overall *Enterococcus* spp. contamination of all locations during the experiment was higher in CE units. In control units, grid floors and drinking nipples seemed critical locations after disinfection. Luyckx, et al. [36] also showed that drinking cups are critical locations for C&D in broiler houses.

A limitation of our study was that the CE protocol was only carried out in pig nursery units, and not in farrowing units. Therefore, the piglets gut microbiota was already formed, which could contain pathogens and contaminate pig nursery units on arrival. Conversely, this is also a drawback of the CE protocol. A future perspective could be to determine the efficacy of a CE protocol applied on the whole farm, however this approach would substantially increase the work load and associated costs for the farmer.

## Conclusions

Very few studies about the impact of microbial cleaning and administration during production on the environment in animal houses are available. Our results showed that competitive exclusion by probiotic-type bacteria could not meet the claims provided by the manufacturer. Moreover, this study showed that a good C&D protocol during down-time is still very important for reducing infection pressure in nursery units. However, more research should be carried out for a valuable alternative, because disinfectant resistance might be an upcoming problem.

## Abbreviations

AC: After cleaning
AD: After disinfection
BC: Before cleaning
BPW: Buffered peptone water
C&D: Cleaning and disinfection
CE: Competitive exclusion
CFU: Colony forming units
CS (%): Proportion of countable samples given in percentage
D (%): Proportion of positive samples after detection given in percentage
*E. coli*: *Escherichia coli*
ILVO: Institute for agricultural and fisheries research
MBC: Minimum bactericidal concentration
MIC: Minimum inhibitory concentration
MRSA ST398: Methicillin resistant *Staphylococcus aureus* sequence type 398
MRSM: chromID® MRSA-SMART medium
PIP AHC: Probiotics in process animal house cleaner
PIP AHS: Probiotics in process animal house stabilizer
Q1: First quartile
Q2: Median
Q3: Third quartile
QAC: Quaternary ammonium compounds
TD100: Treatment days per 100 days at risk
W1: After one week of production
W5: After five weeks of production
